# Pragmatic cluster randomized trial to evaluate effectiveness and implementation of EHR-facilitated collaborative symptom control in cancer (E2C2): addendum

**DOI:** 10.1186/s13063-022-06983-6

**Published:** 2023-01-09

**Authors:** Jeph Herrin, Lila J. Finney Rutten, Kathryn J. Ruddy, Kurt Kroenke, Andrea L. Cheville

**Affiliations:** 1 Yale University School of Medicine, New Haven, CT USA; 2 Medical Affairs, Exact Sciences, Madison, WI USA; 3 Department of Quantitative Health Sciences, Mayo Clinic, Rochester, MN USA; 4 Division of Medical Oncology, Mayo Clinic, Rochester, MN USA; 5 Indiana University School of Medicine, Indianapolis, IN USA; 6 Regenstrief Institute, Inc, Indianapolis, IN USA; 7 Robert D. and Patricia E. Kern Center for the Science of Health Care Delivery, Mayo Clinic, Rochester, MN USA; 8 Department of Physical Medicine and Rehabilitation, Mayo Clinic, Rochester, MN USA

**Keywords:** Electronic health record, Neoplasm, Pain, Palliative care, Patient care team, Patient-reported outcome measure, Quality of life, Self-management, Systems integration, Survivor

## Abstract

We previously described the hypotheses, outcomes, design, and analysis for E2C2, a pragmatic stepped-wedge trial to assess an intervention to improve symptom control in patients with cancer. Subsequent consideration of the design and cohort led to the addition of a second primary hypothesis. This article describes and presents the rationale for this second hypothesis. This addendum also details a revised analytic approach, necessitated by inconsistencies in the original analytic plan. The design, outcomes, and other aspects of the protocol remain unchanged.

## Background

The Enhanced, Electronic Health Record (EHR)-facilitated Cancer Symptom Control (E2C2) is a pragmatic, hybrid type 2 stepped-wedge cluster-randomized clinical trial (SW-CRT) that will assess the impact of an intervention to control Sleep disturbance, Pain, Physical function impairment, Anxiety, Depression, and Energy deficit (SPPADE) symptoms among patients with cancer. The E2C2 trial is designed to assess the impact of the intervention spanning the cure-directed, survivorship, and palliative phases of disease management, while also exploring factors relevant to the intervention implementation. The control condition is the provision of usual care to cancer patients. The original aims of this trial were:

*Aim 1*: To conduct a cluster randomized pragmatic trial with a stepped wedge design to test the hypothesis that a symptom-control–focused, collaborative-care–based E2C2 intervention will significantly reduce SPPADE symptoms, reduce unplanned hospitalizations and visits to the emergency department (ED), improve adherence to cancer therapies, enhance quality of life, and extend survival

*Aim 2*: To evaluate the hypothesis that the use of a multifaceted, evidence-based implementation strategy to support the adoption and use of the E2C2 technologies will improve patient and clinical outcomes

*Aim 3*: To conduct a mixed methods evaluation to detect and identify disparities in the adoption and implementation of the E2C2 intervention among elderly and rural-dwelling patients with cancer, two groups that have had disproportionately more cancer symptoms and worse outcomes

The full protocol for this trial has been previously published [1]. This article documents two important updates to the original protocol. First, subsequent consideration of the study cohort and intervention suggested that aims 1 and 2 should each address two hypotheses, rather than the originally proposed single hypothesis for each. Second, a review of the statistical analysis plan (SAP) identified inconsistencies in the proposed analyses, which has led to a revised analytic plan.

Herein, we describe the additional hypotheses, providing our rationale for inclusion and presenting the updated statistical approach. While we highlight key aspects of the design relevant to the statistical analysis plan, interested readers are encouraged to refer to the original protocol for additional information about the trial [1].

## Primary hypotheses

We originally proposed to address the aims of this study by testing the hypotheses that the intervention (aim 1) improves symptom scores and measures of treatment and (aim 2) improves patient and clinical outcomes. As previously described, the primary outcomes of the study are a bundle of 6 symptom and functioning scores (see below), and the original analysis plan was designed to test for differences in these scores between patients exposed to the intervention when compared with those who are not; similarly, aim 2 entailed testing a hypothesis about differences between intervention groups. The concern regarding these hypothesis tests is that they assess the effect of the intervention across the full cohort of patients enrolled. However, key features of the intervention are only triggered if a patient reports elevated symptom scores and based on a review of baseline data we now anticipate that a large proportion of the cohort will never report elevated symptoms. For these patients with mild or no symptoms, there is both a floor effect—little or no opportunity to improve symptoms—and at the same time no practical exposure to the intervention. While the original hypotheses regarding population-level effects retain primary importance, we think it is of equal importance to test whether the intervention is effective in the subset of patients that ever have elevated symptom scores. Thus, we are revising aims 1 and 2 to include additional specific hypotheses:

*Aim 1*: To conduct a cluster randomized pragmatic trial with a stepped wedge design to test the hypothesis that a symptom-control–focused, collaborative-care–based E2C2 intervention will (primary outcome) significantly reduce SPPADE symptom scores and (secondary outcomes) reduce unplanned hospitalizations and visits to the emergency department (ED), improve adherence to cancer therapies, enhance quality of life, and extend survival. We will test two specific hypotheses:

*H1a (original hypothesis)*. SPPADE symptom scores will improve more, relative to prior scores, for patients who have been exposed to the intervention when compared with the improvement of patients that are not exposed.

*H1b (new hypothesis)*. The effect of the intervention on change in symptom scores will be greater in those patients with at least one prior elevated score.

We will test analogous hypotheses for the secondary outcomes in aim 1 and also for aim 2:

*Aim 2*: To evaluate the hypothesis that the use of a multifaceted, evidence-based implementation strategy to support the adoption and use of the E2C2 technologies will improve patient and clinical outcomes.

*H2a (original hypothesis)*. Patient and clinical outcomes will be better for patients who have been exposed to the intervention when compared to patients that are not exposed.

*H2b (new hypothesis)*. The effect of the intervention on patient and clinical outcomes will be greater in those with at least one prior elevated score.

Aim 3 is unchanged. These additional hypotheses answer questions that are practically relevant; in clinical practice, the more important elements of the intervention would only be offered to the group of patients with at least one elevated SPPADE symptom score. By including these additional primary hypotheses H1b and H2b, we increase the utility of the findings by elevating the reporting of effects that will be of most interest to clinicians and decision-makers.

## Design

### Overview

Briefly, the trial is being undertaken at multiple sites, with clusters defined at the level of the cancer care team; each cluster includes between 2 and 20 oncologists/hematologists and mid-level providers. The trial’s stepped wedge design (SWD) randomized the order of E2C2 implementation among 15 clusters, with 3 clusters randomized to each of 5 sequences. All clusters will be observed for 6 months under the control condition prior to the transition of clusters in the first sequence (step 1). Transitions from control to intervention occur 8 months apart.

This SW-CRT design allows data to be sequentially collected from all clusters for a minimum of 6 months during pre-E2C2 usual care and a minimum of 8 months during the implementation phase. This design also allows us to compare outcomes both across intervention groups and with historical controls at the same clinic, supporting a rigorous assessment of the impact of E2C2 on controlling SPPADE symptoms among patients with cancer while also exploring factors relevant to its implementation across all sites.

There is no transition period between control and intervention steps in a sequence, as it is anticipated that the impact of the intervention will be immediate. Due to the nature of the intervention, blinding was deemed infeasible.

### Cohort

The E2C2 trial is population based; all patients receiving care for solid tumors from the included clinicians at any of the study sites are included in the study sample irrespective of cancer type or stage. At 4 sites, patients with malignant hematology are also included. For aims 1 and 2, we will test separate hypotheses for the main cohort and for the subset of the cohort with at least one elevated symptom score at some point.

### Intervention

The E2C2 intervention includes patient- and clinician-directed elements that are designed to increase the frequency with which patients receive individualized, preference-concordant, and guideline-based care for their symptoms and to increase rates of symptom control. All patients treated by clinicians in clusters in the control state will receive usual care. Additional details of the intervention are previously published [1].

One critical and relevant feature of the intervention is the EHR Triage and Decision Support Algorithms. Patients’ responses to questionnaires will initially assign symptomatic patients to one of 2 incremental levels of stepped care. Patients reporting symptoms 0–3 will be given usual care; those reporting any symptoms 4–6 will be triaged to level 1 intervention (moderate symptoms); and those reporting any symptoms 7–10 will be triaged to level 2 (severe symptoms). We define an “elevated” symptom score as any score greater than 3. Though other criteria also trigger enhanced intervention (non-response to treatment; patient preference for SCM contact; or clinician referral), it is notable that the SPPADE symptom scores used to triage patients are also outcomes, as noted below. It is also notable that patients who never report moderate or severe symptoms will not receive key elements of the intervention.

### Sampling structure

All patients seen by study cancer care teams have primary outcome data collected prior to each visit. In addition, patient visits to clusters that have implemented the intervention are assigned additional questionnaires through the “patient portal,” a web-based tool where patients access information about their visits and medical records. Thus, each patient will have repeated measures over time, with all measures will be attributed to the same cancer care team. In addition, a patient may have visits to a cancer care team during both control and intervention steps of a cluster sequence. In this case, outcome measures will be assigned to the respective treatment status. Patients will enter and leave the trial over the entire study period. Preliminary data suggest the average gap between the first and last date of a patient assessment to be about 50 days, or less than one-fourth the length of each study step.

### Trial status

Baseline data collection began in March 2019 and will continue through January 2023, for a total of 46 months. During the first 42 months of the study, approximately 45,000 patients were included.

### Sample size

The planned sample size is 40,000 patients, which was anticipated to provide almost 100% power to detect a true difference of 0.2 in at least one SSPADE score [1]. A 0.2 difference represents less than 0.1SD for any of the 6 scores. Given the current enrollment of 45,000 patients, we anticipate similar power for the original primary hypothesis. For the new hypothesis, preliminary analyses indicate that 77% of patients, or an anticipated 38,000, will have at least one elevated score, suggesting adequate power to detect similar effects for the smaller cohort.

## Outcomes

### Primary outcomes

The primary outcomes are 5 Sleep disturbance, Pain, Anxiety, Depression, and Energy (SPADE) scores and 1 physical function score (“SPPADE”) [2 – 4]. Patients will complete the 6 SPPADE numerical rating scales (NRSs) prior to medical oncology clinic appointments at all stages of the trial, i.e., prior to and after E2C2 initiation. These electronic patient-reported outcomes (ePROs) will be more frequently administered to symptomatic patients outside of their appointment-linked assessments as part of the intervention. To avoid potential bias from oversampling of symptomatic patients, only NRSs collected in association with clinic appointments will be included in the primary analysis. SPPADE scores are self-reported measures using 0–10 Likert scales to rate the degree of each symptom. The physical function score is also an 11-point scale. The 6 SPPADE scores will be our primary outcomes.

### Secondary outcomes

In addition to our primary outcomes, we will assess secondary outcomes including a composite SPPADE score (the average of all 6 SPPADE NRSs), depression, anxiety, pain, and additional measures of physical function using NRSs. We will also assess the use of healthcare, including hospitalizations, visits to the ED, visits to the outpatient clinic, and calls to the oncology care team. Vital status will also be assessed.

### Randomization

**Fig. 1:**
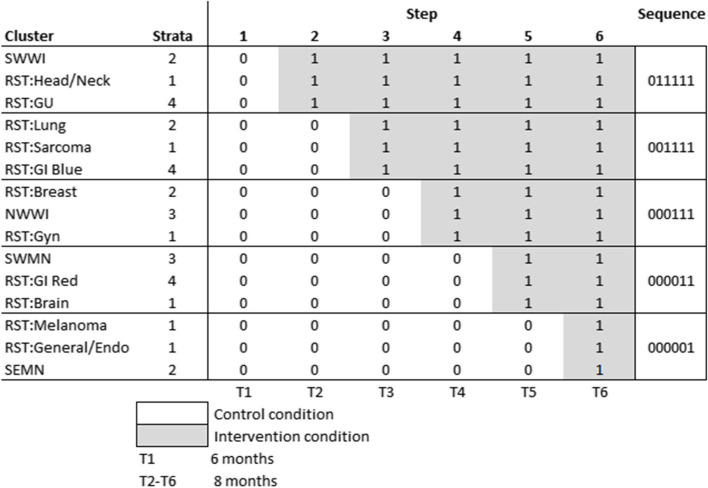
Final allocation of 15 cancer care sites to 5 sequences over 6 time periods

In a stepped-wedge cluster randomized study, clusters are randomized to sequences; each sequence represents a timeline of implementation for clusters in that sequence, with the transition to implementation for clusters in each sequence occurring in a different step. The 15 cancer care teams represented 11 teams at Mayo Clinic Rochester (RST) and 4 Mayo health system community clinics in Southwest Wisconsin [SWWI], Northwest Wisconsin [NWWI], Southwest Minnesota [SWMN], and Southeast Minnesota [SEMN]. These 15 cancer care teams were randomized to 5 sequences, stratified on 3 factors:

- Annual volume, categorized as <4000, 4001–8000, and 8000+ using the calendar year prior to E2C2 initiation
- RST team cancer type: head/neck, genitourinary (GU), lung, sarcoma, gastrointestinal (GI), breast, gynecological, brain, and general/endocrine, and mixed (the 4 community sites)
- RST GI team: Mayo Rochester has 2 GI cancer care teams; internally (and in Fig. 1), they are denoted the “red” and “blue” GI teams

The interactions of these three factors included many empty cells, resulting in only 4 distinct strata. Randomization was performed using a computer program which allocated clusters by strata to sequences. All clusters were randomized by the study statistician (JH) prior to the study start, and the allocations revealed to all clusters at the beginning of the study. The assignment to strata and sequences is illustrated in Fig. 1.

### Additional features

Details on sample size, data collection and management, secondary outcome determination, and implementation assessment were detailed in our prior protocol [1].

### Statistical analyses

What follows represents an updated analytic approach. The original analytic plan described weekly cluster rates as the unit of analysis, while also stating that the effect of the intervention on the weekly rates would be adjusted for patient clinical and demographic factors. Recognizing that these assertions were inconsistent, we have revised the statistical model to one which uses each patient symptom score as the unit of observation. The updated analytic approach also addresses the additional primary hypotheses described above. All methods continue to be appropriate for SW-CRTs and will be performed on an intention-to-treat basis [5]. All analyses will be performed at the conclusion of the study period.

### Aim 1 analyses

The unit of analysis is the patient encounter. We will summarize patient characteristics (e.g., sex, age, cancer type and stage, insurance status) by intervention status (pre-E2C2, E2C2) of the encounter. All patient encounters will be analyzed on an intention-to-treat status; this principle will be extended to the cluster status, so that delays in the implementation of an intervention will not affect the intervention status of patients.

We will include a common set of variables in all models testing the effectiveness of the intervention. Variables capturing cancer type, stage, and treatment status will be included, as well as 6 variables selected as likely to impact the use of the portal and completion of the surveys: from the EHR, we will capture age (categorized into 10-year increments), sex (male or female), and severity of illness (Elixhauser score as a categorical variable). We will also link the patient 9-digit zip code to Census data to get estimates of educational level, income, and broadband access, which will be categorized into quartiles.

### Hypotheses H1a

To test the primary hypothesis H1a for our primary outcomes, we will use generalized linear mixed models to assess the effects of the intervention on each of the 6 SPPADE scores [5]. All models will account for the correlation of outcomes within clusters and across patients and for changes in this correlation over time. We will adjust for time, measured by the study period, to account for temporal effects. Each score will be adjusted for the prior score, with a coefficient that depends on the time *t* since the prior score; to account for potential changes in the correlation of outcomes over time, we will include autoregressive terms at the patient level [6]. To account for changing correlation within clusters over time, we will incorporate a discrete-time correlation structure [7].

Our primary analysis will treat each SPADE score as a continuous variable and employ a mixed effects generalized multivariate model; we will model all 6 scores jointly, which will allow us to account for the correlation between them and avoid correcting for multiple hypothesis tests. Because there is no evidence or prior literature to support a minimally important difference in the SSPADE scores, we will test whether the effects of the intervention on SSPADE scores differ from 0.

Specifically, let *Y*_*ijkm*_ be the *i*th score (*i*=1, … ,6) of the *j*th encounter for the *k*th patient in the *m*th cluster (*m* =1, … ,15), for *j* = 2, … ; the first score for each patient (*j*=1) is a baseline score collected during the first visit, not an outcome. Let *w*=1, … ,6 indicate the trial step during which the survey is collected.

Then, to test the hypothesis that the intervention impacts the 6 symptom scores, we will estimate the joint models:

$${Y}_{ijkm}={A}_{ikm}+{\boldsymbol{B}}_{\boldsymbol{i}}{X}_{jkm}+{\boldsymbol{L}}_{\boldsymbol{i}}{Y}_{i\left[j-1\right] km}+$$

1 $$\boldsymbol F{\boldsymbol Z}_{\mathbf k}\:+\:\boldsymbol D{\boldsymbol S}_{\mathbf m}\:+\:\boldsymbol Qq_{jmk}\;+\;e_{ijkm};\;i=1,\;\dots\;,6$$

where

*X*_*jkm*_ is an intervention indicator which equals 1 if encounter *j* for patient *k* in cluster *m* was post-intervention and 0 otherwise

***B***_***i***_ is a vector of fixed effects for the intervention on symptom score *i*, *i* = 1, … ,6

***L***_***i***_ is a vector representing the effect of the most recent measurement on the current one

***Z***_***k***_ is a vector of patient factors for patient *k*, ***F*** is a corresponding vector of fixed effects

***S***_***m***_ is a cluster-level set of indicators for randomization strata, ***D*** is a vector of fixed effects

***Q*** is a vector representing the secular time effect, where *q*_*jmk*_ are consecutive 2-month time calendar periods

In addition

*A*_*ikm*_ is a vector of random effects, [*A*_1*km*_, … ,*A*_5*km*_] specified as varying across patients nested within discrete quarter-team clusters

*A*_*ikm*_ ~ [*A*_*i*_ + *α*_*ikm*_ + *γ*_imq_ ]; *i*=1,..,6

[*α*_*ikm*_] ~ N(0,∑_*α*_)

[*γ*_*im*_ ] ~ N(0,∑_*γ*_)

and *e*_*ijkm*_ ~ N(0,∑_*e*_) is a vector of residual errors and the ∑s are exchangeable correlation matrices.

Prior to estimating this model, the distributions of the SPPADE scores will be examined and, if appropriate, transformed to have approximately normal distributions. Covariates will be similarly reviewed and either transformed or categorized before inclusion in the mode.

In general, fitting the above models (1) which have several random effects (in both time and across clusters) can be challenging. However, Markov Chain Monte Carlo estimation is very effective at fitting such models and is appropriate for assessing stepped-wedge cluster randomized designs [8–10]. Moreover, by incorporating historical time trends into our priors for the effect of calendar time, a Bayesian model can increase statistical power relative to a frequentist approach [11]. All other priors will be flat. In the event that model (1) cannot be estimated, we will simplify the error structure and other features using a review of variable distributions and correlations, including intra-cluster correlations and time-dependent correlation, to inform the simplifications. Then, using our final model, we will calculate posterior probabilities and credible intervals to assess whether coefficients differ from zero; specifically, we will achieve the primary aim of the study by calculating the posterior probabilities that each of *B*_1_, …, *B*_6_ is greater than 0; we will accept that the intervention is effective if max (PP [abs (*B*_*i*_ ) > 0], *i*=1,..6) ≥ 0.80.

We will estimate models using the Stan software package [12].

### Hypotheses H1b

To test whether the intervention has a differential effect on those patients with at least one elevated symptom score, we will construct an indicator, *E*_*ijkm*_, varying by measurement time, which indicates whether the *i*th prior symptom score was elevated (>3). By interacting these indicators with the main intervention effects ***B***_***i***_, we can assess the impact of the intervention on the corresponding scores of patients with at least one elevated score:

$${Y}_{ijkm}={A}_{ikm}+{\boldsymbol{B}}_{\boldsymbol{i}}{X}_{jkm}+{\boldsymbol{C}E}_{ijkm}+{\boldsymbol{D}}_{\boldsymbol{i}}{E}_{ijkm}{X}_{jkm}+{\boldsymbol{L}}_{\boldsymbol{i}}{Y}_{i\left[j-1\right] km}+$$

2 $$\boldsymbol F{\boldsymbol Z}_{\mathbf k}\:+\:\boldsymbol D{\boldsymbol S}_{\mathbf m}\:+\:\boldsymbol Qq_{jmk}\;+\;e_{ijkm};\;i=1,\;\dots\;,6$$

We will then test the hypothesis as above by calculating max (PP [Abs (D_i_)>0], *i*=1, … ,6) ≥ 0.80.

### Secondary and sensitivity analyses

In a secondary analysis, we will classify each symptom score into low (0–6) or high (7–10) and estimate a model similar to (1) and (2) using a logit link. In sensitivity analyses, we will replicate the main analyses where models (1) and (2) have been modified to include (a) time as a calendar week, implemented with splines; (b) an interaction between time (measured by *q*_*jmk*_ in the notation above) and intervention; and (c) an interaction between calendar time since the intervention and intervention status.

### Secondary outcomes

For secondary outcomes of unplanned hospitalization or ED visit, therapy adherence, quality of life, and survival, we will estimate generalized linear models analogous to (1) but with a single dependent variable (i.e., non-multivariate) and appropriate link functions.

To address a secondary objective of this aim, to better understand the effectiveness of components of the E2C2 intervention in reducing symptoms, we will perform a separate set of analyses including only the intervention patients to assess the impact of specific process measures on SPADE scores and other clinical outcomes. For each process measure, we will first examine the distribution of values (frequency or duration) and, if highly skewed, categorize into 2 or more categories. We will then estimate for each outcome a mixed effects linear model similar to (1) above, though with a single dependent variable. By testing for an overall process measure effect, we can assess which processes contribute most to reducing outcomes; we will also report measures of variance explained to enable relative comparison of process measure effects.

For all models, we will report measures of fit such as predictive *R*^2^ values and variance estimates, as well as convergence measures for Markov Chain Monte Carlo (MCMC) estimation.

### Specific aim 2 analyses

The analysis for aim 2, which is restricted to only intervention encounters, is updated only to include the same patient factors included in aim 1. For the evaluation of the implementation bundle, we will summarize patient characteristics by intervention status (baseline and intervention). We will also summarize all survey responses by cluster implementation status, including clinician characteristics (age, sex, and years of practice). As with aim 1, analyses will be based on intention to treat at the patient level and at the cluster level.

We will assess the effect of process measures on SPADE scores and other clinical outcomes. To assess the effect of clinician experience on implementation processes, we will assess the relationship between clinician scores and implementation outcomes by modeling each outcome as dependent on clinician scores. For count data, we will use appropriate models and select standard or zero-inflated Poisson or negative binomial models according to the Akaike information criterion. For binary measures, we will use logit models. For each outcome, we will estimate bivariate models, including only one survey score, a model with all scores, and a final model that also includes clinician characteristics. These models will include the patient factors included in model (1): cancer type and stage, as well as 7 variables selected as likely to impact the use of the portal and completion of the surveys: age, sex, and severity of illness (Elixhauser), and, according to a 9-digit zip code, area estimates of educational level, income, and broadband access.

We will use the measures of adoption, fidelity, and penetration (described above) to assess the differential impact of E2C2 components on patient and clinician outcomes. Formally, we will undertake a mediation analysis with structural equation modeling to assess the extent to which each component of E2C2 mediates the intervention. We will use models appropriate for count data, selecting standard or zero-inflated Poisson or negative binomial models according to Aikake’s Information Criteria, or logit models for binary measures. For each outcome, we will estimate (a) bivariate models including only one survey score, (b) a model with all scores, and (c) a final model which also includes clinician characteristics.

### Specific aim 3 analyses

Analyses for this aim will be analogous to those for aim 2, though restricted to elderly and rural-dwelling patients.

## Summary

This statistical analysis plan updates our previously published protocol for the E2C2 trial, a stepped-wedge cluster randomized trial to assess the effect of an intervention to improve symptoms in cancer patients. There are two significant changes. First, this new plan adds a second main hypothesis to the first aim, to assess the impact of the intervention on those patients for which there is at least one elevated symptom score. Second, this new plan addresses inconsistencies in the original statistical plan by supporting the adjustment for patient-level covariates, while increasing statistical power. This revised plan has been approved by the Mayo Clinic Institutional Review Board as an update to the originally approved protocol and has been finalized prior to the completion of trial enrollment and data collection.

## Data Availability

Given concerns about confidentiality and waiver of consent, datasets generated for the present study will not be made publicly available. Access to the full final protocol, deidentified data, and statistical analysis will be made available from the study principal investigator upon reasonable request after the publication of primary trial results. The protocol described herein was registered with ClinicalTrials.gov on 25 March 2019 (No. NCT03892967). Primary findings from this study will be reported to ClinicalTrials.gov.
